# Sensory Re-Weighting in Human Bipedal Postural Control: The Effects of Experimentally-Induced Plantar Pain

**DOI:** 10.1371/journal.pone.0065510

**Published:** 2013-06-26

**Authors:** Antoine Pradels, Didier Pradon, Petra Hlavačková, Bruno Diot, Nicolas Vuillerme

**Affiliations:** 1 Université Grenoble-Alpes, FRE 3405 AGIM Laboratory, CNRS-UJF-UPMF-EPHE, La Tronche, France; 2 Université Versailles Saint Quentin en Yvelines, EA 4497, CIC-IT 805, APHP Service de physiologie et d'exploration fonctionnelle, Hôpital Raymond Poincaré, Garches, France; 3 Centre de Podologie de l'Estacade, Grenoble, France; 4 Hôpital Couple Enfant, CHU de Grenoble, France; 5 IDS, Montceau-les-Mines, France; 6 Institut Universitaire de France, Paris, France; Charité University Medicine Berlin, Germany

## Abstract

The present study was designed to assess the effects of experimentally-induced plantar pain on the displacement of centre of foot pressure during unperturbed upright stance in different sensory conditions of availability and/or reliability of visual input and somatosensory input from the vestibular system and neck. To achieve this goal, fourteen young healthy adults were asked to stand as still as possible in three sensory conditions: (1) No-vision, (2) Vision, and (3) No-vision – Head tilted backward, during two experimental conditions: (1) a No-pain condition, and (2) a condition when a painful stimulation was applied to the plantar surfaces of both feet (Plantar-pain condition). Centre of foot pressure (CoP) displacements were recorded using a force platform. Results showed that (1) experimentally-induced plantar pain increased CoP displacements in the absence of vision (No-vision condition), (2) this deleterious effect was more accentuated when somatosensory information from the vestibular and neck was altered (No-vision – Head tilted backward condition) and (3) this deleterious effect was suppressed when visual information was available (Vision condition). From a fundamental point of view, these results lend support to the sensory re-weighting hypothesis whereby the central nervous system dynamically and selectively adjusts the relative contributions of sensory inputs (i.e. the sensory weightings) in order to maintain balance when one or more sensory channels are altered by the task (novel or challenging), environmental or individual conditions. From a clinical point of view, the present findings further suggest that prevention and treatment of plantar pain may be relevant for the preservation or improvement of balance control, particularly in situations (or individuals) in which information provided by the visual, neck proprioceptive and vestibular systems is unavailable or disrupted.

## Introduction

The plantar aspect of the foot is the first point of contact between the body and the external environment in standing, thus providing crucial sensory information to the central nervous system through cutaneous afferent feedback for the control of upright posture (e.g., [1], [2]). Indeed, plantar cutaneous mechanoreceptors provide detailed spatial and temporal information about contact pressures under the foot and shear forces resulting from body movement that constitute valuable feedback to the postural control system. This has even led previous researchers to consider the plantar surface as a “dynamometric map for human balance control” [1]. Since cutaneous feedback from the plantar surface may be influenced by the interaction of the foot with the ground, repeatedly changing the characteristics of the supporting surface experimentally has been reported to modify the control of bipedal posture. Our group recently evaluated the effect of a painful mechanical plantar stimulation on the control of unperturbed bipedal posture [3]. In this study [3], ten young healthy adults were asked to stand upright, eyes closed, as still as possible in three experimental conditions: (1) a no pain condition, (2) a condition when a painful stimulation was mechanically applied to the plantar surfaces of both feet and (3) a condition in which a painful stimulation was applied to another body part not involved in the control of bipedal posture, the palms of both hands. The results showed that, for the same perceived intensity of pain, the painful stimulation applied to the plantar surfaces of both feet increased the displacement of the centre of foot pressure, whereas the painful stimulation applied to the palms of both hands did not. A painful stimulation targeted at parts of the body or systems which are involved in the performance of a postural task interferes with the postural control mechanisms, whereas a painful stimulation of parts of the body or systems which are not involved in the performance of the postural task does not. These results suggest that experimentally-induced pain of the plantar aspect of the foot mainly affects the control of unperturbed bipedal stance via sensorimotor rather than cognitive processes. It is important to note that this experiment was performed in the absence of visual information and in a condition of accurate and reliable vestibular and somatosensory information from the neck. Indeed, considering the importance of vision (e.g., [4], [5]) and neck and vestibular information on the control of bipedal posture (e.g., [6]–[9]), it was important that these factors were controlled in order to specifically assess the potential postural effects of experimental plantar pain. It is however well-established that humans use a variety of sensory sources, including visual, vestibular and proprioceptive sources, to control unperturbed bipedal posture (e.g., [8], [10]–[12]). What is more, in order to adapt to novel and changing conditions within the environment, the individual and the task, and to preserve optimal postural control when one or more sensory channels are degraded, the central nervous system can selectively and dynamically reweight different sensory modalities by: (1) decreasing its reliance (or weight) when sensory information from one or more sensory systems is unavailable or becomes unreliable, and/or (2) increasing its reliance on alternative available sensory inputs that provide accurate and reliable information for controlling posture. This adaptive mechanism has been referred to as the “sensory re-weighting hypothesis” of human postural control (e.g., [10], [13]–[16]). Whether sensory re-weighting in the control of unperturbed bipedal stance is modified by experimentally-induced plantar pain is yet to be established. Determining this is important from both fundamental and applied points of view for the development of interventions aimed at improving postural stability and avoiding falls in patients suffering from plantar pain.

The present study was designed to address this issue, by investigating the effects of experimentally-induced plantar pain on the displacement of centre of foot pressure during unperturbed bipedal stance in different sensory conditions. The different conditions related to the availability of visual information and the reliability of somatosensory information from the vestibular system and neck. It was hypothesized that that (1) experimentally-induced plantar pain would increase CoP displacements in the absence of vision [3] (hypothesis 1), (2) this deleterious effect would be accentuated when somatosensory information from the vestibular system and neck was altered (hypothesis 2), and (3) this deleterious effect would be suppressed when visual information was available (hypothesis 3).

## Results

### Perceived pain intensity

Analysis of the visual analogue scores showed a main effect of Pain (F (1,13)  = 962.31, *P*<0.001). All subjects reported no pain for the No-pain conditions. For the Pantar-pain conditions, the mean visual analogue scores were 6.7±0.9 cm, 6.9±0.8 cm and 6.8±0.9 cm, for the Vision, No-vision and No-vision – Head tilted backward conditions, respectively. There were no differences in the visual analogue scores between sensory conditions (*P*>0.05). All subjects described the mechanical stimulation as a distinct pricking pain confined to a small area around the site of stimulation without spread.

### Postural analyses

#### Surface area covered by the trajectory of the CoP

**Figure 1:**
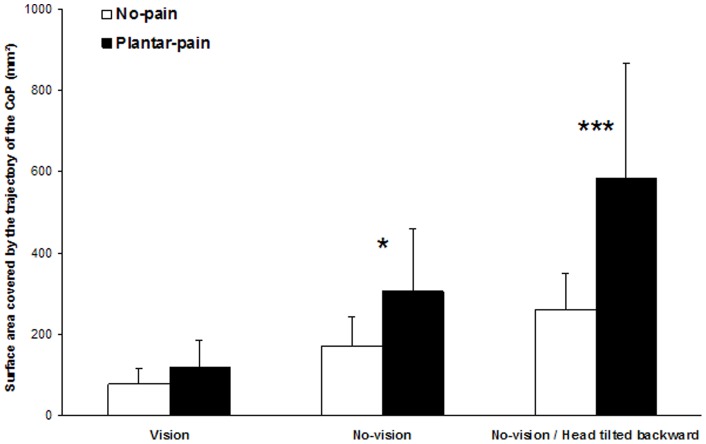
Mean and standard deviation of the surface area covered by the trajectory of the CoP obtained for the three sensory conditions and the two pain conditions. The No-pain condition is represented by *white bars* and the Plantar-pain condition by *black bars*. The significant *P* values for comparison between No-pain and Plantar-pain conditions are reported (*: *P*<0.05; ***: *P*<0.001).

Analysis of the surface area covered by the trajectory of the CoP showed main effects of the Pain (*F* (1,13)  = 19.93, *P*<0.001) and Sensory conditions (*F* (2,26)  = 44.85, *P*<0.001) and a significant interaction between the conditions (*F* (2,26)  = 15.43, *P*<0.001). Further analysis of this interaction (Figure 1) indicated that:

1. the Plantar-pain condition produced a non significant change in the CoP surface area relative to the No-pain condition in the Vision condition (*P*>0.05),
2. the Plantar-pain condition produced an increased CoP surface area relative to the No-pain condition in the No-vision condition (*P*<0.05),
3. this effect of Plantar-pain was more accentuated in the No-vision – Head tilted backward condition than in the No-vision condition (*P*<0.001).

#### Standard deviation of the CoP displacements along the ML axis

**Figure 2:**
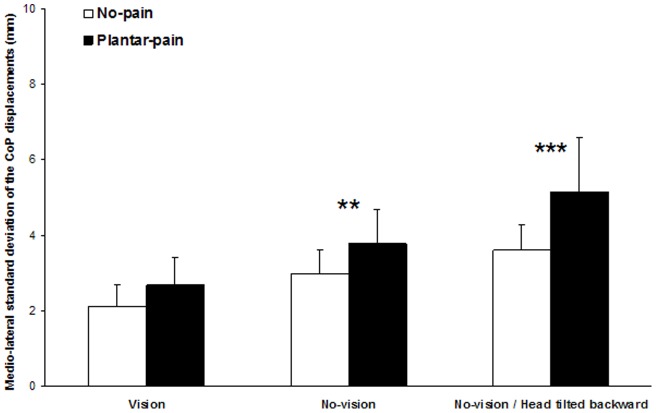
Mean and standard deviation of the standard deviation of the CoP displacements along the medio-lateral axis for the three sensory conditions and the two pain conditions. The No-pain condition is represented by *white bars* and the Plantar-pain condition by *black bars*. The significant *P* values for comparison between No pain and Pain conditions are reported (**: *P*<0.01; ***: *P*<0.001).

Analysis of the standard deviation of the CoP displacements along the ML axis showed main effects of Pain (*F* (1,13)  = 27.95, *P*<0.001) and Sensory conditions (*F* (2,26)  = 41.08, *P*<0.001) and a significant interaction between the Pain and Sensory conditions (*F* (2,26)  = 8.37, *P*<0.01). Further analysis of this interaction (Figure 2) indicated that:

1. the Plantar-pain condition produced a non significant change of the standard deviation of the CoP displacements along the ML axis relative to the No-pain condition in the Vision condition (*P*>0.05),
2. the Plantar-pain condition produced an increased standard deviation of the CoP displacements along the ML axis relative to the No-pain condition in the No-vision condition (*P*<0.01),
3. this effect of Plantar-pain was more accentuated in the No-vision – Head tilted backward condition than in the No-vision condition (*P*<0.001).

#### Standard deviation of the CoP displacements along the AP axis

**Figure 3:**
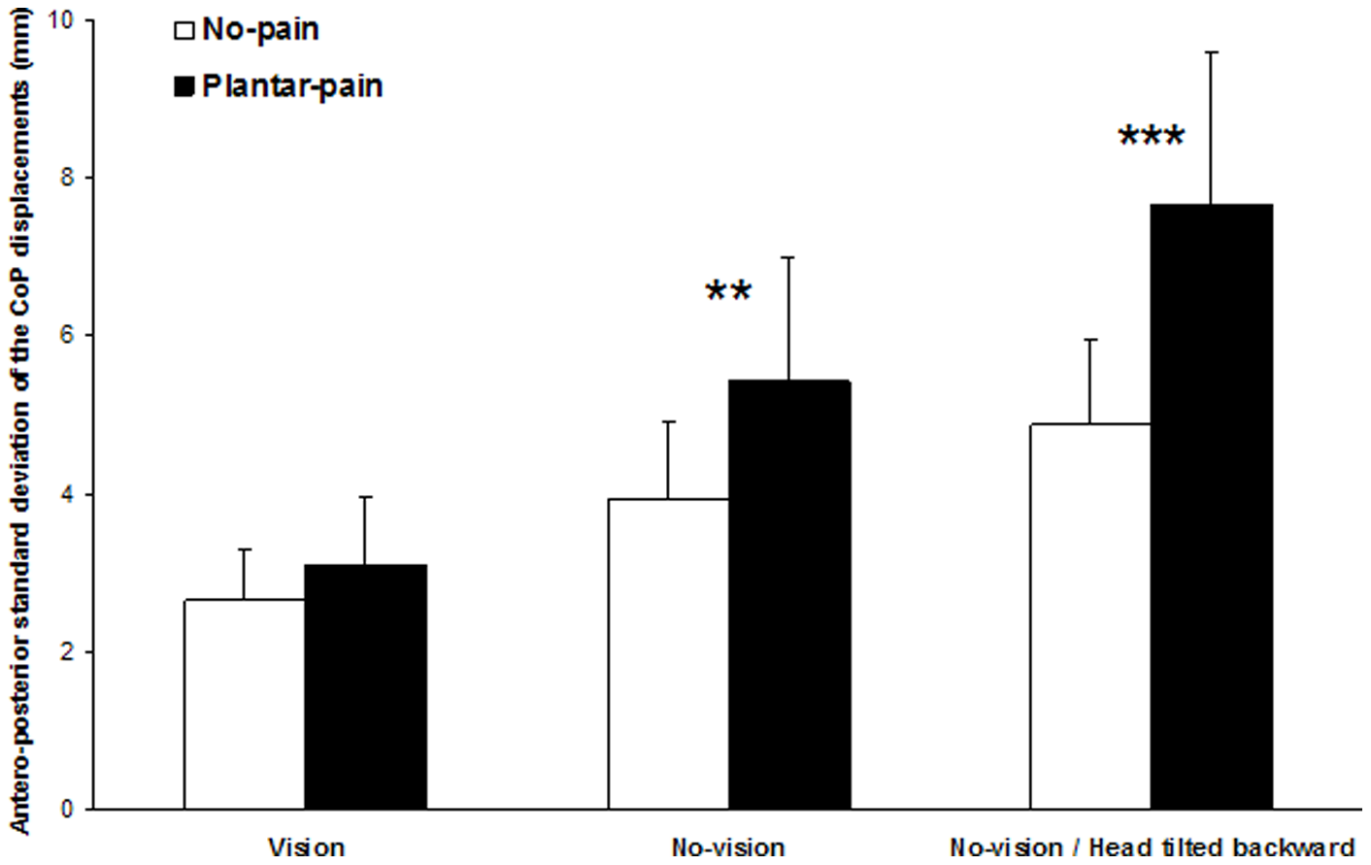
Mean and standard deviation of the standard deviation of the CoP displacements along the antero-posterior axis for the three sensory conditions and the two pain conditions. The No-pain condition is represented by *white bars* and the Plantar-pain condition by *black bars*. The significant *P* values for comparison between No-pain and Plantar-pain conditions are reported (**: *P*<0.01; ***: *P*<0.001).

Analysis of the standard deviation of the CoP displacements along the ML axis showed main effects of the Pain (*F* (1,13)  = 24.21, *P*<0.001) and Sensory conditions (*F* (2,26)  = 58.97, *P*<0.001) and a significant interaction between the conditions (*F* (2,26)  = 18.83, *P*<0.001). Further analysis of this interaction (Figure 3) indicated that:

1. the Plantar-pain condition produced a non significant change in the standard deviation of the CoP displacements along the AP axis relative to the No-pain condition in the Vision condition (*P*>0.05),
2. the Plantar-pain condition produced an increased standard deviation of the CoP displacements along the AP axis relative to the No-pain condition in the No-vision condition (*P*<0.01),
3. this effect of Plantar-pain was more accentuated in the No-vision – Head tilted backward condition than in the No-vision condition (*P*<0.001).

## Discussion

We investigated the effects of experimentally-induced pain on the plantar surfaces of the feet on the displacement of centre of foot pressure during unperturbed upright stance in different sensory conditions of availability of visual information and reliability of somatosensory information from the vestibular system and neck. To achieve this goal, fourteen young healthy adults were asked to stand as still as possible in three sensory conditions: (1) No-vision, (2) Vision and (3) No-vision – Head tilted backward, carried out in two experimental conditions: (1) a No-pain condition and (2) a condition when a painful stimulation was applied to the plantar surfaces of both feet (Plantar-pain condition) [3]. Centre of foot pressure (CoP) displacements were recorded using a force platform.

When visual information was unavailable (No-vision condition), the results showed that experimentally-induced plantar pain increased CoP displacements. This result was expected (hypothesis 1), in line with a recent study that used a similar experimental protocol to induce experimental pain on the soles of both feet [3]. At this point, a potential limitation of the present study pertains to the experimental design used in the present experiment to induce pain of the plantar soles. It is indeed possible that the pyramids used in the Plantar-pain condition of the present experiment may have had a mechanical effect (e.g., modification of the base of the foot) that led to increased CoP displacements observed in this experimental condition relative to the No-pain condition. To address this issue, a control experiment was performed on six young healthy adults to assess if a non-painful plantar mechanical stimulation also increased CoP displacements during unperturbed upright stance in the absence of visual information. To achieve this goal, two 1-cm rigid square pyramid shapes, identical to those used in the present experiment except that the point was not sharp were used to experimentally induce the non-painful mechanical stimulation. These objects were placed at the same location than those used in the Plantar-pain condition. Results showed that the non-painful plantar mechanical stimulation resulted in decreased CoP displacements. The findings of this control experiment hence suggested that the increased CoP displacements observed in the Plantar-pain condition relative to the No-pain condition in the present study did not stem from the mechanical modification of the base of the foot associated with our experimental design [3].

More originally, the results of the present study showed that the effects of experimentally-induced plantar pain depended on the sensory environment. Indeed, the destabilizing effect of experimentally-induced plantar pain which occurred in the absence of vision (No-vision condition) was accentuated when somatosensory information from the vestibular system and neck was altered by asking the subjects to tilt their head backwards in the sagittal plane (No-vision – Head tilted backward condition), and was reduced when visual information was available (Vision condition). These two results confirm hypotheses 2 and 3, respectively. Taken together, they suggest an increased reliance on somatosensory inputs from the vestibular system and neck as well as visual information in order to control unperturbed bipedal stance in the presence of plantar pain. From a fundamental point of view, these results lend support to the sensory re-weighting hypothesis (e.g., [10], [13]–[16]) whereby the central nervous system dynamically and selectively adjusts the relative contributions of sensory inputs (i.e. the sensory weightings) in order to maintain balance when one or more sensory channels are altered by the task (novel or challenging), environmental or individual conditions. Examples of the ability of the postural control system to adapt to constraints acting on the individual have been provided in previous studies assessing the effects of plantar-flexor muscle fatigue on the control of bipedal stance in unaltered and altered sensory environments. These studies have demonstrated the ability of the central nervous system to (1) *down-weight* proprioceptive cues from the ankle [17], [18]) which are degraded by the fatiguing exercise [19], [20] and (2) *up-weight* visual information [17], [21], somatosensory inputs from the foot and ankle [22]–[24], somatosensory information from the vestibular system and neck [25] and haptic cues from the finger [26]. The up-weighting of these afferent signals provides more accurate and reliable information to ensure adequate bipedal postural control in a condition of plantar–flexor muscle fatigue. With regard to the hypothesis of the modification of plantar sensory input induced by experimentally-induced pain, our results are consistent with those obtained when somatosensory information from the plantar surface was degraded by the interposition of a foam support surface beneath the feet. Previous studies have reported that the destabilizing effect of standing on a compliant foam support on the control of unperturbed bipedal posture was more pronounced with the head extended rather than in a neutral position (i.e., in a condition of altered proprioceptive information from the vestibular system and neck) [27]–[30]. This effect was significantly reduced when visual information was provided [4], [30], [31]. To our knowledge, such sensory reweighting mechanisms in conditions of experimentally-induced plantar pain have not been demonstrated before. At this point, it is possible that the ability to use visual information to compensate for the destabilizing effect induced by experimentally-induced plantar pain could depend on the pain intensity, but also on the physical and physiological visual parameters (e.g. visual acuity, central and peripheral visual fields). In the present experiment, the experimentally-induced pain was perceived as severe (the mean visual analogue scores were 6.7±0.9 cm, 6.9±0.8 cm and 6.8±0.9 cm, for the Vision, No-vision and No-vision – Head tilted backward conditions, respectively) and the visual parameters have been controlled (subjects with normal or corrected-to-normal visual acuity were instructed to look at the intersection of a black cross (20 cm ×25 cm) positioned on a white wall at a distance of 1 m in front of them, at eye level). Along theses lines, whether, with vision, the magnitude of the effect of experimentally-induced plantar pain on the control of unperturbed upright stance depends on the intensity of the painful stimulation is currently being assessed in different visual conditions.

Finally, in addition and complementary to their relevance in the field of neuroscience, the present findings also could have clinical applications. The results of this study could certainly lead to the hypothesis that plantar pain does represent a factor which increases postural instability and risk of falls, especially when the postural control system is concomitantly challenged by sensory manipulations. From a clinical point of view, we suggest that prevention and/or treatment of plantar pain may be very important for the preservation or improvement of balance control, particularly in situations (or individuals) in which the information provided by the visual, neck proprioceptive and vestibular systems is unavailable or disrupted (as occurs daily, when moving from a bright to a dark environment, when looking or reaching to a cupboard above eye level, or in the case of systemic diseases). Clinical trials are currently underway in order to address this issue.

## Materials and Methods

### Subjects

Fourteen young healthy adults (age: 23.4±4.4 years; body weight: 66.1±8.6 kg; height: 172.9±6.7 cm; mean ± SD) voluntarily participated in the experiment. Subjects had to be healthy without a history of neck pain, neurological or vestibular impairment, injury or operation in the cervical spine and normal or corrected-to-normal visual acuity. The study was conducted in accordance with the Declaration of Helsinki and was approved by the national ethics committee (French society for independent-living technologies and gerontechnology). Participants gave their informed written consent to the experimental procedure.

### Task and procedure

**Figure 4:**
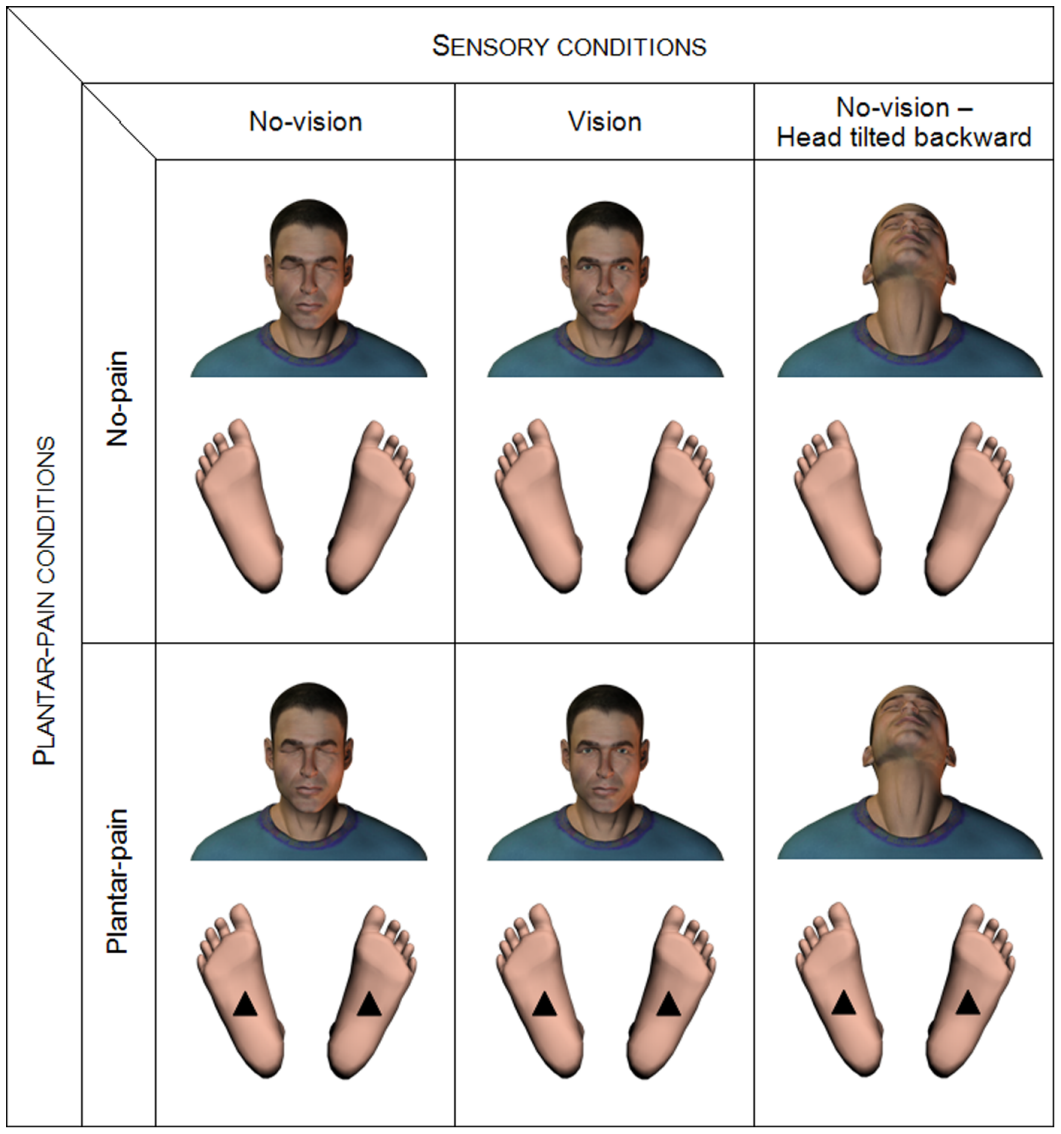
Illustration of the six experimental conditions used the study.

Subjects stood barefoot on a force platform (Dynatronic, France; sampling frequency 40Hz), feet abducted to 30°, heels 3-cm apart, arms hanging loosely by their sides. They were asked to stand as still as possible [32] in three sensory conditions: (1) No-vision, (2) Vision and (3) No-vision – Head tilted backward (Figure 4). In the No-vision condition, subjects were asked to close their eyes and to keep their head facing straight ahead. In the Vision condition, they were asked to stare at the intersection of a black cross (20 cm ×25 cm) positioned on a white wall at a distance of 1 m in front of them, at eye level [21] In the No-vision – Head tilted backward, subjects were asked to close their eyes and to tilt their head backwards to at least 45° in the sagittal plane (e.g., [27]–[29], [33]–[38]). The Head tilted backward posture is recognised to induce (1) a modification of the orientation of the vestibular organs that places the utricular otoliths well beyond their working range and renders balance related vestibular information unreliable to the central nervous system (e.g., [33], [38], [39], [40]) and (2) abnormal sensory inputs from neck proprioceptors (e.g., [39], [41], [42]), which represents a challenge for the postural control system. Subjects adopted the required head-neck posture 10 s prior to the initiation of data collection and maintained the posture for the duration of the trial. The experimenter stood by the subjects to monitor their posture and their head position throughout the trial.

These three sensory conditions were performed in two experimental conditions: No-pain and Plantar-pain (Figure 1). The No-pain condition served as a control condition. In the Plantar-pain condition, two 1-cm rigid square pyramid shapes (polyurethane, hardness shore A110), were used to experimentally induce the painful stimulation, similarly to the protocol recently used in a study that evaluated the effects of experimentally-induced plantar pain on centre of foot pressure displacements during unperturbed upright stance [3]. More precisely, in order to apply the pain on the soles of the feet, one rigid pyramid was placed, under each foot, at the center of the plantar surface at the intersection between a a line drawn through the center of the heel and the second metatarsal, and a transversal line that divides the plantar surface (but not the toe) in two equal parts (see [3], Figure 1).

For each sensory condition of Vision, No-vision and No-vision – Head tilted backward and each experimental condition of No-pain and Plantar-pain, subjects performed three 30-s trials. The order of the six experimental conditions was randomized. Rest periods of 60-s were provided between each trial during which subjects were allowed to sit down. The repeatability of foot placement between trials was ensured by drawing the outline of the feet on the top of the force platform.

### Pain intensity assessment

At the end of each trial, subjects were asked to rate their perception of the pain induced by the stimulation by drawing a line on a 10 cm visual analog scale where 0 cm indicated “no pain” and 10 cm “intolerable pain”. Visual analogue scales have been validated for experimental pain [43].

### Balance assessment

Three dependent variables were used to describe subject's postural behaviour: (1) the surface area (mm^2^) of the trajectory covered by the CoP which represents a measure of the spatial variability; (2) the standard deviation of the plantar CoP displacements along the medio-lateral (ML) axis and (3) the standard deviation of the plantar CoP displacements along the antero-posterior (AP) axes (mm). The results of a recent study established the test–retest reliability of these three CoP-based parameters as “excellent” with three 30 s trial recordings [44].

### Statistical analysis

For each dependent variable, a 2 Pain (No-pain *vs.* Plantar-pain) ×3 Sensory conditions (Vision *vs.* No-vision *vs.* No-vision – Head tilted backward) analyses of variance (ANOVAs) with repeated measures on both factors were performed. Post-hoc analyses (*Scheffe*'*s Test*) were used whenever necessary. Level of significance was set at 0.05.
